# Production of a Recombinant Non-Hydroxylated Gelatin Mimetic in *Pichia pastoris* for Biomedical Applications

**DOI:** 10.3390/jfb10030039

**Published:** 2019-09-02

**Authors:** Pia Gellermann, Caroline Schneider-Barthold, Svenja Nicolin Bolten, Ethan Overfelt, Thomas Scheper, Iliyana Pepelanova

**Affiliations:** Institute of Technical Chemistry, Leibniz University Hannover, Callinstraße 5, 30167 Hannover, Germany

**Keywords:** gelatin-mimetic protein, recombinant gelatin, *Komagataella pfaffi* (*Pichia pastoris*), recombinant ECM-protein

## Abstract

Proteins derived from the natural extracellular matrix like collagen or gelatin are common in clinical research, where they are prized for their biocompatibility and bioactivity. Cells are able to adhere, grow and remodel scaffolds based on these materials. Usually, collagen and gelatin are sourced from animal material, risking pathogenic transmission and inconsistent batch-to-batch product quality. A recombinant production in yeast circumvents these disadvantages by ensuring production with a reproducible quality in animal-component-free media. A gelatin mimetic protein, based on the alpha chain of human collagen I, was cloned in *Pichia pastoris* under the control of the methanol-inducible alcohol oxidase (AOX1) promoter. A producing clone was selected and cultivated at the 30 L scale. The protein was secreted into the cultivation medium and the final yield was 3.4 g·L^−1^. Purification of the target was performed directly from the cell-free medium by size exclusion chromatography. The gelatin mimetic protein was tested in cell culture for biocompatibility and for promoting cell adhesion. It supported cell growth and its performance was indistinguishable from animal-derived gelatin. The gelatin-mimetic protein represents a swift strategy to produce recombinant and human-based extracellular matrix proteins for various biomedical applications.

## 1. Introduction

Collagen and its denatured derivatives, such as gelatin, are used for many biomedical applications including wound dressings, graft coatings, suture material, drug release, and tissue engineering [1,2,3]. These materials, despite their wide use, possess several disadvantages associated with their origin and method of production. Collagen and its derivatives are sourced from animal tissue, which brings the risk of allergic reactions, as well as the transmission of pathogens like viruses and prions. The age and physiological state of the tissue of origin brings further variability to the final collagen product. Gelatin is produced from denatured collagen by acidic or alkaline extraction [4]. This commercial process delivers wide variation in batch-to-batch product quality, which can represent a challenge for medical applications [5].

The production of recombinant collagen-based materials allows the circumvention of the above-mentioned drawbacks associated with animal-derived materials. Therefore, many groups have aimed to produce recombinant collagen-based materials in various organisms. Recombinant collagen I has already been produced in transgenic mice, tobacco plants, silk worms, insect cell lines, and mammalian cell lines [5]. A production in simple microorganisms like yeast and bacteria would make a recombinant collagen mimetic protein more attractive in terms of achievable yields and production costs [6]. However, the complexity of helical collagens makes it challenging to integrate them into a microbial recombinant system [7].

Type I collagen, the most commonly used collagen type in biomedical applications, is a heterotrimer consisting of two alpha 1 and one alpha 2 chains. The central helical domain of this molecule consists of repetitive Gly-Xaa-Yaa sequences, with Xaa and Yaa being proline and hydroxyproline respectively. The thermal stability of collagen is dependent on the hydroxylation of proline with the enzyme prolyl-4-hydroxylase (P4H), which is present in sufficient amounts only in mammalian cell expression systems. In order to produce correctly-folded collagen in most expression systems like plants, insect cells, bacteria, and yeasts, it is therefore necessary to also clone both subunits of the P4H enzyme. The group of Myllyharju accomplished the successful cloning, production and expression of human collagen types I, II and III in the yeast *Pichia pastoris* (*P. pastoris*) [8]. To achieve this, the enzyme P4H was also cloned into the yeast strains. The authors could show that the recombinant collagens are triple-helical, but as a result, cannot be exported from the yeast cell [9]. The recombinant collagen from *P. pastoris* has been successfully commercialized [10].

In this study, we aimed to produce a recombinant collagen fragment which can be secreted from the cell into the media and be produced in simple microorganisms using animal component-free media. Also, we wanted to circumvent the production of P4H and the cloning of its associated subunits, but still obtain a gelling product. The cloning of collagen fragments into *Escherichia coli* (*E. coli*) or yeast without P4H results in the production of random-coiled collagens, which resemble gelatin in their structure. Therefore, in this work we present the production of a non-hydroxylated gelatin-mimetic protein (GelMP) in *P. pastoris*. We selected this yeast, as it is known that yeasts are able to convert repetitive genes better than *E. coli* and because we were aiming for extracellular production [11]. Our goal was to design a cost-efficient, simple and quick process starting from the cloning procedure to the final purification of the material. In order to simplify the process, we cloned a single gene coding for a 400 amino acid segment from the helical region of the human collagen I alpha1 chain and included repetitive prolyl-glycyl-prolyl (PGP)-sequences flanking on both sides of the collagen sequence. Such artificial repeats are inspired by the Gly-Xaa-Yaa structure of collagen and are known to possess thermal trimerization capacity under specific circumstances [12]. The group of Werten et al. demonstrated that gels from these telechelic triblock (ABA) protein polymers are formed on long incubation times at appropriate concentrations, in accordance with studies performed on synthetic (Pro-Gly-Pro)_9_ peptides (PGP) [13]. In our study, we combined a sequence from the helical region of collagen I to introduce bioactivity and PGP repeats to incorporate gelling behavior without hydroxylation by P4H.

## 2. Results and Discussion

### 2.1. Production of GelMP

**Figure 1:**
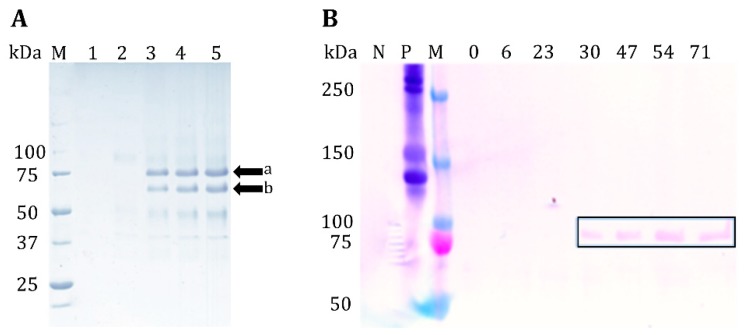
(**A**) SDS-PAGE of cultivation supernatant samples, 1: glycerol phase before induction at 48 h of cultivation; 2: Induction with methanol t = 71 h; 3: 72 h; 4: 78 h; 5: 80 h. M: Marker Precision Plus Protein™ Unstained Protein Standard. (**B**) Western blot of supernatant, N: negative control; P: human collagen I as a positive control; M: Prestained protein ladder, 0–71 = cultivation hours; in this cultivation methanol induction was performed earlier at t = 29 h. GelMP can be observed 1 h after induction at t = 30 h.

A 1.9 kb fragment, encoding the 40.7 kDa GelMP, was cloned into the *P. pastoris* expression vector pPIC9K. The vector thus obtained was used to transform *P. pastoris* GS115. Successful vector integration was confirmed using colony PCR (data not shown). Several Mut+ transformants were randomly chosen and tested for GelMP production in shaking flasks by methanol induction. A representative GelMP transformant was selected for fermentation experiments. Culture supernatants harvested throughout the fermentation were analyzed with SDS-PAGE (Figure 1A).

After 1 h of induction with methanol the target protein can be observed at 75 kDa (Figure 1A, lane 3). SDS-PAGE analysis of intracellular fractions of the cultivation revealed that GelMP does not accumulate inside the cell (please see Supplementary Material, Figure S1). The identity of the secreted protein was confirmed by both Western blot (Figure 1B) and mass spectrometry (data not shown). The observed molecular weight of ca. 75 kDa in the gel electrophoresis is higher than the theoretical molecular weight of 40.7 kDa. It has already been widely described in the literature that collagen-like proteins and synthetic gelatins migrate at an apparent molecular weight one to four times higher than their true molecular weight in SDS-PAGE gels [14,15]. An additional explanation for the higher observed molecular weight might be glycosylation of the protein. This hypothesis was tested with a PNGase F assay and was found to be negative (data not shown). Furthermore, mass spectrometry analysis did not reveal any glycosylation as well, leading to the conclusion that the protein is truly 40.7 kDa, but appears larger due to erratic behavior in SDS-PAGE.

Apart from the full-length band at 75 kDa, another band of lower molecular weight (ca. 65 kDa) was also observed. Both bands were analyzed by N-terminal sequencing and proven to be fragments of the major GelMP protein. Edman sequencing showed that band (a) in Figure 1 is the expected amino acid sequence of the GelMP protein (YVEFPGPPGP), while band (b) corresponds to a lesser fragment of the GelMP protein starting with (GFPGPKGAAG). The smaller GelMP fragment is most likely produced by proteolytic degradation. Indeed, the random-coil structure of unhydroxylated collagens makes them very susceptible to proteolytic degradation [11]. Cultivations strategies for reducing proteolysis like the addition of casamino acids to the culture medium or lower pH values were also tried in efforts to suppress the production of the smaller fragment, but without success [16]. The proteolytic degradation occurs probably already inside the cell. Another strategy for preventing proteolytic degradation would include changing the expression host to a protease deficient strain like SMD1168 or a change in the amino acid sequence of GelMP, making the protein immune to intracellular proteolytic degradation.

### 2.2. Purification of the GelMP Protein

**Figure 2:**
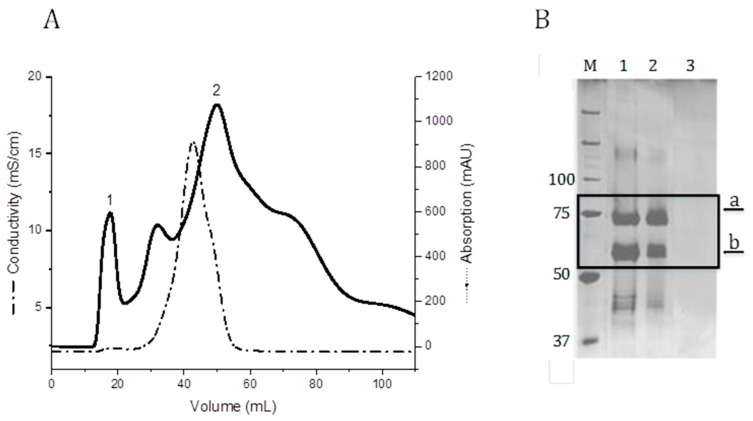
(**A**) SEC using a Sephadex G-25 column (flow rate: 5 mL min^−1^; sample volume: 15 mL). Buffer exchange to phosphate-buffered saline buffer (PBS). 1: Elution peak of GelMP. (**B**) SDS-PAGE analysis of GelMP protein purification (SEC). M: Marker Precision Plus Protein™ Unstained Protein Standards, lane 1: supernatant broth; lane 2: protein after SEC; lane 3: salt peak (2) from Figure 2A.

After cultivation completion, the biomass was removed by centrifugation and the medium containing the secreted protein was processed by chromatography. GelMP was purified directly from the fermentation broth using size exclusion chromatography (SEC). Single-step purification of GelMP with SEC was selected due to there being only a small amount of impurities besides the target protein and its major degradation fragment in the fermentation supernatant (Figure 1). The elution of the SEC is shown in Figure 2A. The target fraction with GelMP can be found in the first peak (1). The second peak (2) contains only culture residues and salts (Figure 2B, lane 3). This is also indicated by the increase in conductivity (Figure 2B). It was not possible to achieve separation between the GelMP protein and its major fragment, so that both proteins were isolated in the same fraction. Downstream efforts to separate the two fragments by fractional precipitation or alternative chromatographic methods did not lead to the desired outcome either. The two proteins possess only a small difference in molecular mass (40.7 kDa to 34.2 kDa) and have highly identical properties making chromatographic separation very challenging. However, since the GelMP fragment does not impair the functionality of the protein as a gelatin mimetic, both proteins were purified together and used for cell culture experimentation. All yield values are thus based on the product GelMP and its major fragment.

The endotoxin value of the isolated product is a critical parameter for biomedical applications. After the SEC, a polishing step using membrane anion-exchange chromatography was introduced, in order to remove any endotoxins present in the material. After this treatment, the endotoxin value was 1.2 EU/mg. Further reduction to this value can be achieved by using pyrogen-free glassware and endotoxin-free water in all process steps.

### 2.3. Structural Characterization

**Figure 3:**
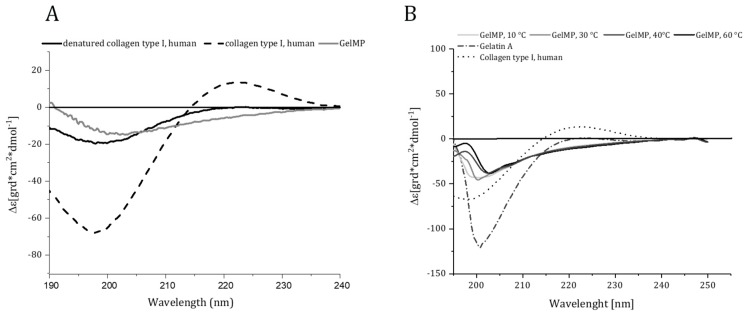
(**A**) Circular dichroism spectra of human collagen I, denatured collagen I and recombinant GelMP. (**B**) Circular dichroism spectra of recombinant GelMP (10–60 °C), gelatin A and human collagen I. Far ultraviolet (190–240 nm) CD spectra were recorded at a concentration of 1 mg·mL^−1^ for all proteins.

CD spectra were recorded for GelMP, human collagen I, heat-denatured collagen, and gelatin. Only human collagen I showed positive ellipticity at λ = 225 nm and a minimum below 200 nm, indicating the presence of a triple helical conformation (Figure 3A). As expected, GelMP did not show this characteristic spectrum at any temperature tested (Figure 3B). The PGP repeats and the non-hydroxylated collagen middle block result in random coil configuration of the protein. Similar findings were obtained by Werten et al., who designed a triblock design protein based on PGP repeats and a random middle block, which also did not display positive ellipticity in the λ = 215 − 240 nm region [12]. Collagen-mimetics based on PGP-repeats can form triple helices at low temperatures and prolonged incubation times. This propensity is greatly increased if stabilizing features like a foldon domain [13] or a mimetic cystine knot [17] are introduced into the protein design.

**Figure 4:**
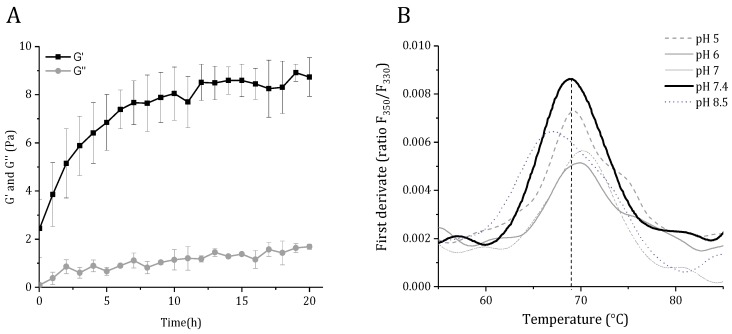
(**A**) Storage (G’) and loss (G’’) moduli for a 2.5 mM GelMP solution. Gel formation was analyzed with a time-sweep experiment (frequency (f) = 1 Hz and strain (γ) = 0.1%). (**B**) Thermal denaturation of GelMP, determined by differential scanning fluorimetry.

The recombinant GelMP behaves like a gelatin mimetic by forming a weak gel on prolonged incubation at temperatures below 37 °C (Figure 4A). The storage modulus was higher than the loss modulus from the start of incubation, indicating the formation of a viscoelastic solid. As a next step, the effect of buffer composition on protein stability was investigated using nano-differential scanning fluorimetry (Figure 4B). The highest apparent melting temperature was observed in PBS buffer at pH 7.4 (Tm = 69 °C).

The observed apparent melting temperature is higher than the one (Tm = 42 °C) reported for triblock protein polymers with (PGP)_9_ repeats, of similar molecular size and using differential scanning calorimetry (DSC) [12]. However, care must be taken when comparing the values, as it is not always possible to directly compare between DSC and differential scanning fluorimetry (DSF) transition temperatures. Here, additional factors such as the thermal scan rate should be considered [18].

### 2.4. Biocompatibility and Functionality of GelMP/Caspase Apoptosis Assay

**Figure 5:**
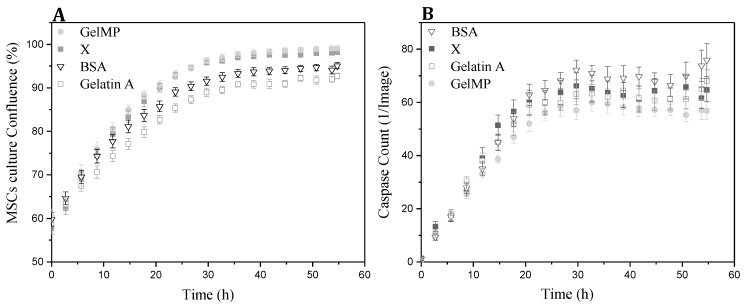
(**A**) Real-time cell confluence study in a hAD-MSCs cell line, media was treated with different additives (GelMP, bovine serum albumin (BSA), gelatin A and no addition (X) as a control). (**B**) The number of caspase-3/7 positive cells was recorded over time and plotted as fluorescence objects, n = 3 wells per data point shown; X = control.

In order to study the potential of GelMP in replacing mammalian derivatives, it was important to investigate its non-toxicity and biocompatibility. hAD-MSCs were cultured with GelMP, gelatin and bovine serum albumin (BSA) at the same concentration in an IncuCyte^®^ Live-Cell Analysis System. The kinetic monitoring of cytotoxicity and apoptosis of the cells was performed in the presence of the Caspase 3/7 Apoptosis Assay Reagent. Images were collected every 2 h. After a 60 h incubation period, images and quantification of apoptosis (green object count/image) showed that GelMP, gelatin and BSA-treated cells (Figure 5B) are all similar to each other and display no toxicity to hAD-MSCs. The recombinant GelMP functions as a typical natural extracellular matrix protein-component (ECM) and is non-toxic to cells.

**Figure 6:**
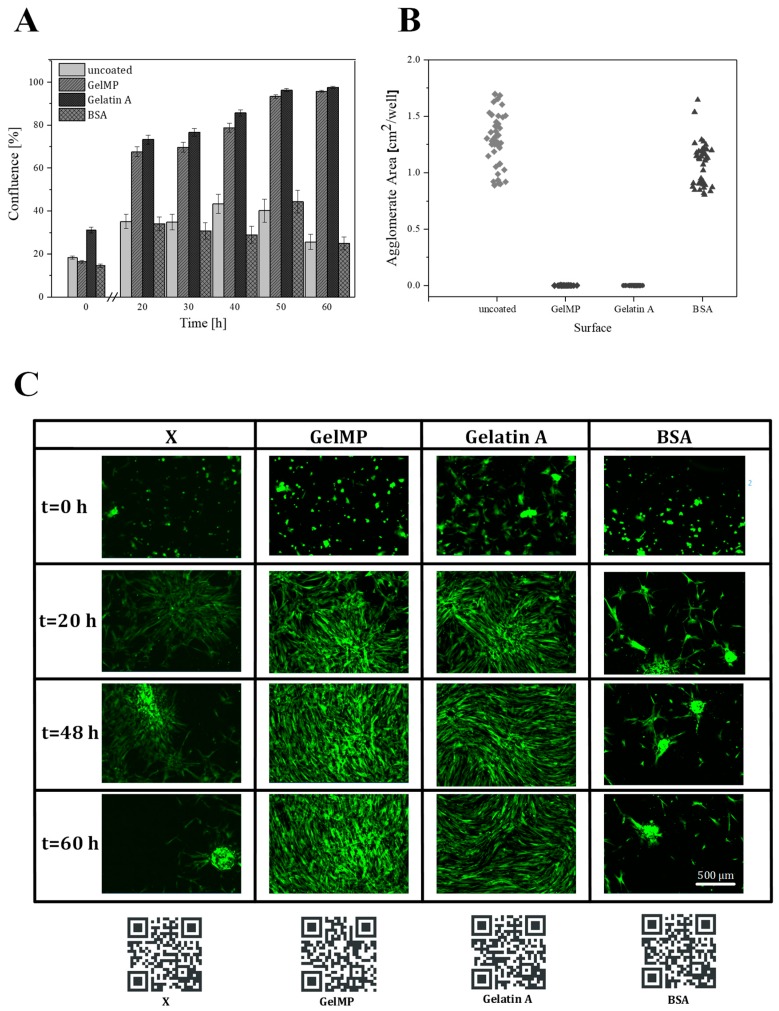
(**A**) Real-time cell confluence study in a hAD-MSCs cell line; growth on four different surfaces (GelMP, gelatin, BSA and X = uncoated surface). The cell population was monitored for 60 h using the IncuCyte^®^ system in an incubator (5% CO_2_ and 37 °C). (**B**) Agglomerate formation on different surfaces. (**C**) Cell morphology on GelMP, gelatin, BSA and X = uncoated surfaces at 0, 20, 48, and 60 h. Scale bar 500 µm. Videos of the cell growth and adhesion process can be found in the supplementary section of the journal and are also linked as a QR-Code.

Adhesion to the extracellular matrix or to other cells is essential for the growth, communication and survival of mammalian cells [19]. For the adhesion assay, the attachment and proliferation of hAD-MSCs to different cell culture coatings was monitored in real-time using the IncuCyte^®^ microscope. As shown in Figure 6, GelMP increased cell confluence in comparison to cell growth on the BSA substrate or plastic cell culture surface alone. By the end of the 20 h culture period, the hAD-MSC cultures in wells coated with GelMP or gelatin demonstrated level of confluence almost double the one exhibited by cultures growing on uncoated surfaces. Gelatin and GelMP show a standard growth curve with up to 100% cell confluence level by 60 h, while cultures growing on the uncoated and BSA-coated wells reach 45% cell confluence (Figure 6A). In addition, MSCs growing on GelMP and gelatin displayed similar elongated and spindle-like morphology (Figure 6C). In addition, hAD-MSCs growing on BSA and uncoated surfaces could not adhere efficiently to the surface and tended to form agglomerates (Figure 6B). The biofunctionality experiments demonstrated that the recombinant GelMP is non-toxic and biocompatible and its performance is indistinguishable to that of animal-derived materials like gelatin.

## 3. Materials and Methods

### 3.1. Vector Construction

**Figure 7:**
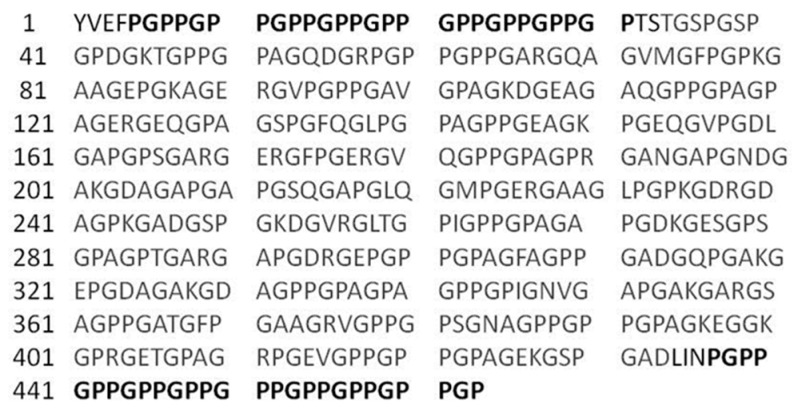
Sequence of the GelMP protein with PGP repeats shown in dark and the collagen sequence displayed in dark grey.

The recombinant GelMP consists of two segments: a 400 amino acid sequence sourced from the alpha chain of type I human collagen (amino acid sequence 541 to 940), and (Pro-Gly-Pro)_9_ (PGP) repeats flanking this sequence on each side (Figure 7). Cleavage sites for SpeI and PacI were inserted between the collagen sequence and the PGP repeats. In addition, an EcoRI and NotI site were inserted at the 5′ and 3′-ends, respectively. The GelMP designed in this manner was codon-optimized and produced by a gene synthesis service (Thermo Fischer, Waltham, MA, USA). The synthetic gene was delivered in a pMK-RQ vector construct. Utilizing the added restrictions sites EcoRI and NotI, the construct was cloned in frame into the cloning/expression region of pPIC9K (InvitrogenTM, Carslbad, CA, USA). The sequence was verified by automated DNA sequencing (Eurofins Genomics, Ebersberg, Germany).

### 3.2. Organism and Transformation

The expression vector pPIC9K-GelMP was linearized with SalI and used to transform competent GS115 (his4) cells according to the protocol of Lin-Cereghino [20]. Transformants were spread on minimal dextrose plates and incubated for 72 h at 30 °C. Clones were screened using colony PCR and potential Mut+ transformants were identified. Recombinants were then tested for multiple insertion of the gene-of-interest on geneticin-containing yeast extract–peptone–dextrose plates (range of G418: 0.5 to 4 mg·mL^−1^). Selected clones growing on the 4 mg mL^−1^ G418 plate were screened for protein expression in shake flasks as described by ref. [21]. The highest producer was selected for further work and transferred to a bioreactor for scale up of production.

### 3.3. Fermentative Production of GelMP in P. pastoris

A 30 L stainless steel bench top bioreactor (Biostat^®^ Cplus, Sartorius, Göttingen, Germany) was equipped with a dissolved oxygen electrode (InPro6820, Mettler-Toledo, Columbus, OH, USA), a pH-electrode (EasyFerm Plus K8 200, Hamilton, Martinsried, Germany), a methanol sensor system (Raven Bio-Tech Inc., Canada), and a peristaltic pump (Ismatec Reglo ICC, Wertheim Germany). A Biostat^®^ Cplus control unit (Sartorius, Göttingen, Germany) was connected to the bioreactor for fermentation control. The methanol sensor system was connected to a single-board computer for data processing. Bioreactor operation data was logged using ProfiSignal (Delphin Technology, Germany). A 1 mL aliquot of a cryo culture (OD_600_ = 50) was thawed and placed in 100 mL medium (per liter: 25 g (NaPO_3_)_6_, 0.93 g CaSO_4_, 18.2 g K_2_SO_4_, 14.9 g MgSO_4_·7H_2_O, 9 g (NH_4_)_2_SO_4_, 40 g glycerol and 0.435% PTM1 (per liter: 6 g CuSO_4_·5H_2_O, 0.08 g NaI, 3 g MnSO_4_·H_2_O, 0.2 g Na_2_MoO_4_·2H_2_O, 0.02 g H_3_BO_3_, 0.5 g CoCl_2_, 20 g ZnCl_2_, 65 g FeSO_4_·7H_2_O, 0.2 g Biotin, 5 mL H_2_SO_4_). The pre-culture was cultivated until the cells reached OD_600_ = 10–15. The bioreactor was filled with 10 L fermentation medium (per liter: 25 g (NaPO_3_)_6_, 0.93 g CaSO_4_, 18.2 g K_2_SO_4_, 14.9 g MgSO_4_·7H_2_O, 9 g (NH_4_)_2_SO_4_, 120 g glycerol and 0.435% PTM1) and was inoculated with cells from the pre-culture to an OD_600_ = 0.1. The operation parameters were pH 5.0, 30 °C, dissolved oxygen (DO) level of 30% air saturation and a gas flow rate of 2L·min^−1^. The DO level was controlled using the stirrer cascade system of the BIOSTAT^®^ Cplus and by supplying pure oxygen if necessary. After depletion of glycerol, the methanol feed (100% methanol with 12 mL·L^−1^ PTM1) was set up to 0.2% v/v methanol. The cultivation ended after 80 h.

### 3.4. Purification of GelMP from the *P. pastoris* Culture Supernatant

The supernatant was harvested after 80 h of cultivation by centrifugation (5000× *g*, 30 min, 4 °C) and stored at −20 °C until further processing. GelMP was purified directly from the fermentation broth using an ÄKTA Pure system (GE Healthcare, Chicago, IL, USA). Then 15 mL of supernatant was loaded on a Sephadex G-25 column (HiPrep™ 26/10 Desalting, GE Healthcare) previously equilibrated with PBS puffer (pH 7.4). GelMP protein was eluted with PBS buffer at a flow rate of 5 mL min^−1^ for 40 min monitoring UV-absorption and conductivity. The peaks eluted from the column were analyzed by 10% sodium dodecyl sulphate-polyacrylamide gel electrophoresis (SDS-PAGE). Fractions of 8 mL were collected and pooled for lyophilization.

A polishing step for the removal of potential endotoxins was also introduced. The target protein was dissolved in Tris-HCl (pH 7.5, 20 mM) to a concentration of 1 mg/mL and was passed through an anion exchange membrane in a flow-through mode (Sartobind^®^ pico, membrane area: 2.9 cm^−2^, membrane volume: 0.08 mL, Sartorius, Göttingen, Germany). The endotoxin concentration after the polishing step was measured with an Endosafe^®^ nexgen-PTS™ system (Charles River, Wilmington, MA, USA).

### 3.5. Bicinchoninic Acid Protein Assay

For the determination of protein concentration, a bicinchoninic acid (BCA) assay (Pierce BCA Protein Assay Kit, Thermo Fisher Scientific, Waltham, MA, USA) was performed. Native collagen I from human placenta (Santa Cruz Biotech., Dallas, TX, USA) was used as a reference.

### 3.6. SDS PAGE and Western Blot Analysis

Protein samples were analyzed with SDS-PAGE using 10% Tris-HCl gels and a Mini Protean Tetra Cell System (BioRad, USA). The culture supernatant was mixed 1:1 with Laemmli buffer (2×) and 10 µL were loaded onto the gel. A Precision Plus Protein Standard (BioRad, Hercules, CA, USA) was used for the determination of molecular weight. Proteins were visualized with Coomassie Brilliant Blue G 250 staining. SDS-PAGE protein bands were blotted onto a PVDF membrane [22], which was then subjected to Western blot analysis using the Western Breeze Immunodetection Kit (Invitrogen, USA) according to the manufacturer’s instructions and using a collagen-I-antibody (Acris Antibodies, Nr. R1038, Herford, Germany). N-terminal sequencing was performed in the Helmholtz Zentrum (HZI) Braunschweig.

### 3.7. Circular Dichroism Spectroscopy

Circular dichroism (CD)-spectra were recorded using a spectropolarimeter J-815 (Jasco, Pfungstadt, Germany). The purified GelMP and gelatin A (Sigma Aldrich, St. Louis, MO, USA) were dissolved in PBS buffer at a concentration of 1 mg/mL. The human collagen I (Biotechnology Inc., USA) was dissolved in acetic acid (1 mg/mL) and was also treated at 90 °C for 10 min for thermal denaturation. Test solutions were placed in a cuvette with a 1 mm path length and spectra were recorded in the 190–240 nm region. The spectra were obtained with a scanning speed of 50 nm/min at a resolution of 0.5 nm. Melting curves from 10 to 60 °C were derived by increasing the temperature in steps of 10 °C. Measurements were performed 5 times and the averaged scan values were plotted.

### 3.8. Label-Free Differential Scanning Fluorimetry

Thermostability was measured by nano differential scanning fluorimetry (DSF) on a Tycho NT 6 instrument (NanoTemper Technologies, Munich, Germany) with a back-reflection aggregation detection at a range from 20–95 °C and with a heating rate of 30 °C/min. Protein unfolding was followed by tryptophan/tyrosine fluorescence intensity at 330 and 350 nm in various buffers covering pH 5–8.5. The apparent melting temperature (Tm) was determined by detecting the maximum of the first derivative of the fluorescence ratios (F350/F330) after fitting experimental data with a polynomial function.

### 3.9. Rheological Characterization

The gel formation of a 2.5 mM GelMP solution in PBS (pH = 7.4) was investigated at 15 °C using a MCR 302 Modular Rheometer, Anton Paar, Austria) equipped with a plate-plate geometry (20 mm diameter, 1 mm gap size). The trimerization was recorded with a time sweep oscillatory test under constant strain amplitude of 0.1% and at a constant frequency of 1 Hz for a period of 20 h. A solvent trap was used to prevent sample loss by evaporation.

### 3.10. Cell Culture and Proliferation

Cell proliferation on GelMP coatings was assessed by using a culture of human adipose-derived mesenchymal stem cells (hAD-MSC). hAD-MSCs were expanded in alpha-MEM medium (Thermo Fisher Scientific, Braunschweig, Germany) containing 1 g L^−1^ glucose, 2 mM L-glutamine, 10% human serum (CC-pro, Oberdorla, Germany) and 50 µg·mL^−1^ gentamicin (Biochrom, Berlin, Germany), harvested by accutase treatment (Sigma Aldrich, St. Louis, MO, USA). Experiments were performed with hAD-MSCs of passages two to twelve.

### 3.11. Cell Adhesion Experiments

The lyophilized GelMP was dissolved in PBS-buffer (pH 7.4) at a concentration of 2 mg mL^−1^. Then 400 µL of this GelMP solution was added in each well and incubated at 4 °C for 12 h to allow protein adsorption to the well surfaces. Next, wells were rinsed with several milliliters of PBS buffer to remove excess protein. As control for the adhesion experiment, a set of wells were coated with bovine serum albumin (BSA) and gelatin (Sigma Aldrich, gelatin type A, St. Louis, MO, USA) with the same concentration and using the same procedure as for GelMP.

For adhesion experiments, hAD-MSCs were seeded onto a coated 48-well-plate (Eppendorf, Germany) at a density of 15,000 cells/cm^2^ with 300 µL medium per well. After seeding, cells were cultivated for 60 h in alpha-MEM containing 10% fetal bovine serum (FBS) and 50 µg mL^−1^ gentamicin in a humidified atmosphere containing 5% CO_2_ and 21% O_2_ at 37 °C. During the cultivation, microscopic pictures were taken every 2 h (four fields imaged per well) using an IncuCyte^®^ Live-Cell Imaging System (Sartorius, Göttingen, Germany). Data were analyzed using the IncuCyte^®^ Confluence software (IncuCyte^®^ S3 2018 A), which quantified cell surface area coverage as confluence values.

### 3.12. Biocompatibility of GelMP by a Quantitative Assay of Caspase-3/7 Kinetic Activation

The hAD-MSCs were seeded in 48 well-plates at a density of 15,000 cells/well in 300 µL culture medium (alpha-MEM containing 10% FBS, 50 µg mL^−1^ gentamicin, and 0.5 µM IncuCyte^®^ Caspase-3/7 reagent). The medium was previously mixed with GelMP, gelatin and BSA, resulting in a final concentration of 5 mg·mL^−1^. A quantitative analysis of apoptosis of hAD-MSCs over time was performed during the cultivation in treated medium (GelMP, BSA, gelatin) and with regular cell culture medium serving as a control. Once treated, the cells were immediately placed inside the IncuCyte^®^ Live-Cell Analysis System (Sartorius Stedim Biotech GmbH, Göttingen, Germany) with a 10× objective in a standard cell culture incubator and both phase-contrast and fluorescent images were collected every 2 h.

## 4. Conclusions

Large-scale production of a recombinant non-hydroxylated gelatin mimetic protein was achieved by methanol-fed cultivation of *P. pastoris*. This yeast is a suitable host for the high-level production of recombinant gelatins. These proteins serve as recombinant extracellular matrix constituents and possess excellent biocompatibility, without risks of pathogenic transmission associated with animal-derived products. The target protein GelMP was produced routinely at levels of >3 g·L^−1^, which is comparable to other recombinant protein production efforts in this yeast. The GelMP was secreted into the media from which it could be directly isolated by SEC. The protein serves as an excellent substrate in cell culture and can support cell attachment and spreading in a manner non distinguishable from animal-derivatives.

## Supplementary Materials

The following are available online at https://www.mdpi.com/2079-4983/10/3/39/s1, Video S1: X. Video S2: GelMP, Video S3: BSA, Video S4: gelatin A, FigureS1: Western Blot of pellet.
