# Supplementary figures and images for: Production of a Recombinant Non-Hydroxylated Gelatin Mimetic in Pichia pastoris for Biomedical Applications
Source: J Funct Biomater. 2019 Sep 2;10(3):39. doi: 10.3390/jfb10030039 (PMC6787575; doi:10.3390/jfb10030039)

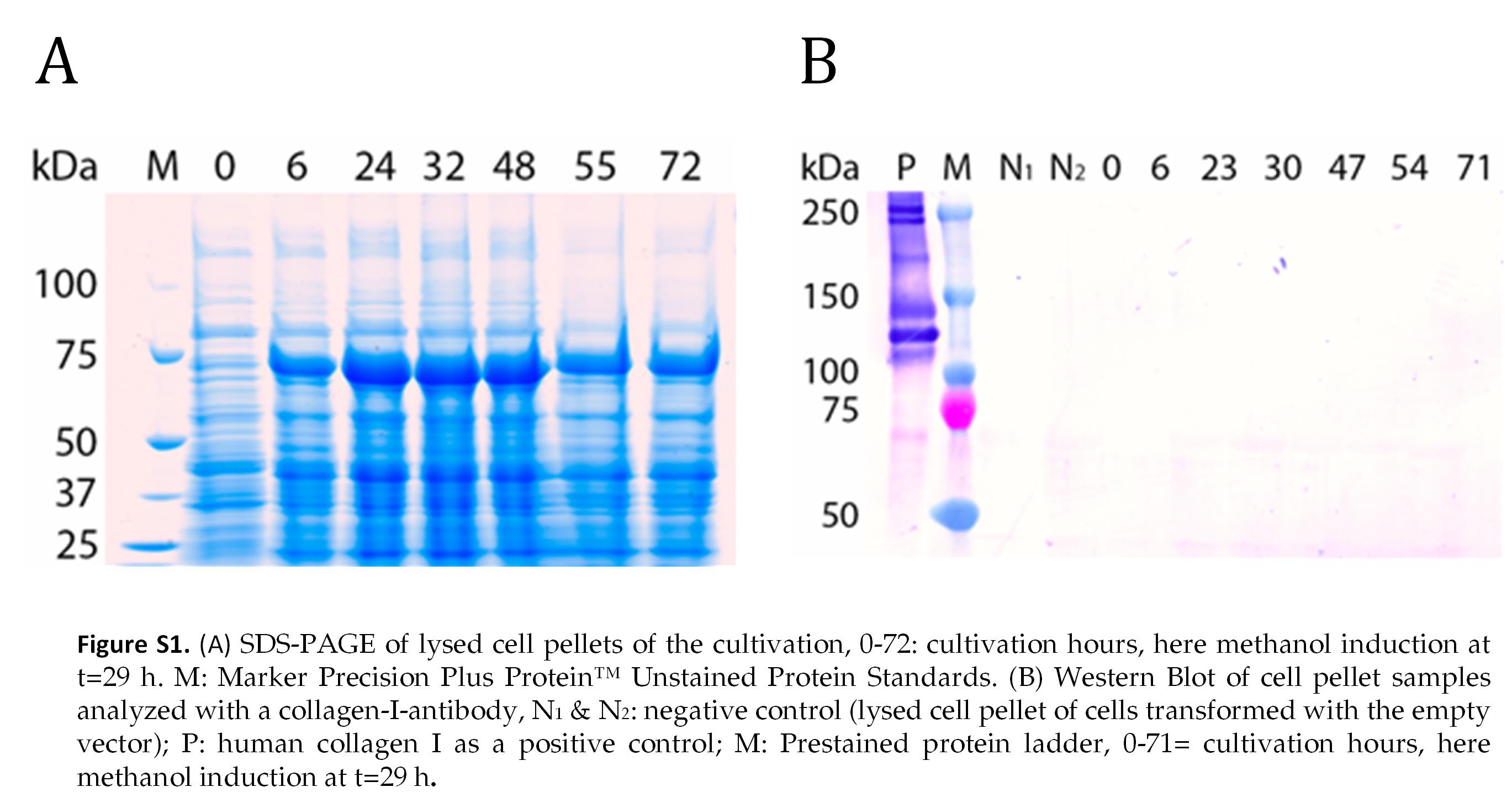

Supplement: Supplementary file 1 [file jfb-10-00039-s001.zip › Supplemtary_files/FigureS1_SDS-PAGE_WesternBlot.jpg]
